# Benefits of PDE5 inhibition in patients with diastolic heart failure

**DOI:** 10.1186/1471-2210-11-S1-O6

**Published:** 2011-08-01

**Authors:** Marco Guazzi

**Affiliations:** 1 University of Milano, San Paolo Hospital, Via A. di Rudinì, 8, 20142 Milano, Italy

## 

The occurrence of signs and symptoms of heart failure (HF) with normal left ventricular ejection fraction and predominant anomalies of left ventricular (LV) relaxation and stiffness (i.e. diastolic HF) is increasingly recognized. Notably, in recent years survival rate of systolic HF has improved, whereas the prognosis for diastolic HF remains unchanged. This may be in part attributable to the lack of additional benefits by treatments’ options used as standard care of systolic HF.

PDE5 inhibition is an emerging therapy proposed for HF treatment that enhances NO signaling by increasing the cyclic guanosine monophosphate (cGMP) availability. This pharmacological property seems especially attractive for patients with left ventricular hypertrophy and diastolic HF [1]. In experimental models of failing hearts due to pressure overload and diastolic dysfunction, PDE5 inhibition has been shown to promote a reverse left ventricular (LV) chamber remodeling by modulating LV hypertrophy and fibrosis [2]. Preliminary observations in humans confirm the ability of long-term PDE5 inhibition administration by sildenafil to improve cardiac geometry, LV mass and diastolic function [3].

Recent community based studies [4] have highlighted that a considerable number of patients with diastolic HF present with left-sided Group 2 pulmonary hypertension suggesting a tight pathophysiological relationship between LV hypertrophy, increasing LV filling pressures and abnormalities of pulmonary vascular tone and permeability. Interestingly, development of pulmonary hypertension strongly discriminates survival rate.

Although in these patients alterations of many signaling pathways may be unique to the heart or pulmonary vasculature, downregulation of the NO-cGMP pathway seems to be a common finding in both locations. Accordingly, in an effort to better understand the combined role of this pathway in both the heart and the lung vasculature, we recently performed a study aimed at testing the hypothesis that diastolic HF patients at higher risk for the unfavorable upstream effects of an increased left atrial pressure would benefit of chronic PDE5 inhibition with sildenafil. Specifically, we hypothesized that sildenafil would modulate pulmonary vascular tone and RV hemodynamic burden. A significant improvement in pulmonary hemodynamics, right ventricular contractility and chamber dimensions were observed at 6 and 12 months of follow-up. These effects were paralleled by an improvement in quality of life and clinical status [5].

Although the available experimental and clinical data seem promising, further large-scale morbidity and mortality studies are necessary to provide a definitive appreciation onto whether PDE5 inhibition may effectively impact the natural history of HF due to diastolic origin.
